# Genetic variation in Northern Thailand Hill Tribes: origins and relationships with social structure and linguistic differences

**DOI:** 10.1186/1471-2148-7-S2-S12

**Published:** 2007-08-16

**Authors:** Davide Besaggio, Silvia Fuselli, Metawee Srikummool, Jatupol Kampuansai, Loredana Castrì, Chris Tyler-Smith, Mark Seielstad, Daoroong Kangwanpong, Giorgio Bertorelle

**Affiliations:** 1 Dipartimento di Biologia ed Evoluzione, Università di Ferrara, via L. Borsari 46, 44100 Ferrara, Italy; 2 Department of Biology, Faculty of Science, Chiang Mai University, Chiang Mai 50202, Thailand; 3 Dipartimento di Biologia Evoluzionistica e Sperimentale-Sezione di Antropologia, Università di Bologna, via Selmi 3, 40126, Bologna, Italy; 4 Wellcome Trust Genome Campus, Hinxton, Cambridge, CB10 1SA, UK; 5 Department of Genetics and Complex Diseases, Genome Institute of Singapore, 60 Biopolis St., Singapore

## Abstract

**Background:**

Ethnic minorities in Northern Thailand, often referred to as *Hill Tribes*, are considered an ideal model to study the different genetic impact of sex-specific migration rates expected in matrilocal (women remain in their natal villages after the marriage and men move to their wife's village) and patrilocal societies (the opposite is true). Previous studies identified such differences, but little is known about the possible interaction with another cultural factor that may potentially affect genetic diversity, i.e. linguistic differences. In addition, Hill Tribes started to migrate to Thailand in the last centuries from different Northern areas, but the history of these migrations, the level of genetic legacy with their places of origin, and the possible confounding effects related to this migration history in the patterns of genetic diversity, have not been analysed yet. Using both original and published data on the Hill Tribes and several other Asian populations, we focused on all these aspects.

**Results:**

Genetic variation within population at mtDNA is lower in matrilocal, compared to patrilocal, tribes. The opposite is true for Y-chromosome microsatellites within the Sino-Tibetan linguistic family, but Hmong-Mien speaking patrilocal groups have a genetic diversity very similar to the matrilocal samples. Population divergence ranges between 5% and 14% at mtDNA sequences, and between 5% and 36% at Y- chromosomes STRs, and follows the sex-specific differences expected in patrilocal and matrilocal tribes. On the average, about 2 men and 14 women, and 4 men and 4 women, are exchanged in patrilocal and matrilocal tribes every generation, respectively. Most of the Hill Tribes in Thailand seem to preserve a genetic legacy with their likely geographic origin, with children adoption probably affecting this pattern in one tribe.

**Conclusion:**

Overall, the sex specific genetic signature of different postmarital habits of residence in the Hill Tribes is robust. However, specific perturbations related to linguistic differences, population specific traits, and the complex migratory history of these groups, can be identified. Additional studies in different populations are needed, especially to obtain more precise estimates of the migration parameters.

## Background

The mountain slopes of Northern Thailand are occupied by a variety of ethnic minorities, often referred to as *Hill Tribes*. There, in a radius of less than 100 miles from the main city of Chiang Mai, about 500,000 peoples live in more than 3,000 villages located at about 1,000 – 1,500 meters of altitude.

This area, and the human groups inhabiting it, are of special interest for a population genetics study for at least three reasons: i) different tribes have different social habits concerning the postmarital residence choice, with different expectations on the ratio between male and female gene flow; ii) extreme cultural differences are observed, which are expected to enhance the genetic structure even at a micro-geographic scale; iii) all these groups have a relatively recent, but controversial and largely unknown, origin from surrounding countries. Here we investigate all these aspects using maternally (DNA sequences of the mitochondrial first hypervariable region) and paternally (Y-chromosome microsatellites) inherited markers.

Sex-specific differences in migration rates are expected in matrilocal and patrilocal societies. Matrilocality, in fact, implies that women remain in their natal villages after the marriage (and men move to the wife's village), whereas the opposite occurs in patrilocal groups. In other words, migration rates are expected to be female-biased or male-biased (i.e., higher in female or in males) in patrilocal and matrilocal populations, respectively. If so, the genetic structure at Y-chromosome markers should be generally stronger in our species than the structure observed at mtDNA markes, since most human groups are patrilocal. Different genetic studies seemed to confirm this expectation [1-3], even though a recent study [4] suggests that the effect of patrilocality can be identified at local, but not global, geographic scale. Human groups in Northern Thailand represent an ideal model to study this effect at a small geographic scale, since the two different social behaviours, matrilocality and patrilocality, are observed in different Hill Tribes. Previous studies on six tribes showed that mtDNA and Y-chromosome diversity are correlated with postmarital residence pattern [5], and patrilocal tribes also appeared to regulate immigration more tightly than matrilocal tribes [6]. But the possible association with other cultural traits, such as language, was not tested. For example, a patrilocal population may strictly control the male immigration rates from groups speaking different languages or dialects, but be much more permissive with immigrants speaking their language. To our knowledge, only two recent studies investigated the possible interaction between patrilocality/matrilocality social behaviour and other cultural traits, with conflicting results. In Indian endogamous castes, Kumar et al. [7] do not find a significant evidence for the influence of postmarital residence patterns on genetic variation, suggesting that other factors may play a role. On the other hand, Bolnick et al. [8] suggest that the expectation based on classification of populations as patrilocal or matrilocal is robust in Native Americans, at least with respect to the linguistic differences. It seems therefore that considering different aspects that probably affect the patterns and levels of gene flow may further clarify the impact of patrilocality/matrilocality on genetic variation.

Large cultural differences characterize the Hill Tribes in Northern Thailand. Tribes, but also subgroups within tribes, have their own languages (often not mutually intelligible), clothing, ornaments, and religion (although conversion to Christianity by western missionaries is common in some groups). The influence of linguistic barriers on genetic structure has been extensively analysed in human groups [9,10], probably because languages, more than other cultural traits, can be classified in a hierarchical manner. We will therefore consider if and how linguistic affiliation is an additional factor in shaping genetic diversity, within and between tribes, in Northern Thailand.

Finally, we will focus on the genetic origin of the Hill Tribes. As recently shown in a study on the genetic origin of Polynesians [11], the analysis of paternally and maternally inherited markers can be very useful to detect sex-specific contributions during the establishment of a population. Historical sources and/or oral traditions suggest that Hill Tribes populations immigrated in Thailand during the last centuries (but perhaps much earlier), some from the surrounding countries (Laos and Myanmar), and some from more distant regions, such as Tibet or Southern China. However, this hypothesis is strongly debated, also because in several cases the presumed places of origin are no longer inhabited by such tribes, or their obvious relatives. Using a clustering analysis, and two specific databases assembled from several published studies on Asian populations (for mtDNA and Y-chromosome markers, respectively), we will analyse the genetic legacy of the Hill Tribes to better understand their geographic origin.

## Results and discussion

### Genetic variation within and between populations

A different genetic signature is expected in markers transmitted exclusively by one of the two sexes, depending on the postmarital residence pattern. In patrilocal societies, where male gene flow should be reduced, low diversity within groups and large differences between groups are expected at Y-chromosome markers, with the opposite pattern predicted at mtDNA markers. On the other hand, if men move between populations and women tend to stay in their birthplace, as in matrilocal populations, the reverse pattern is expected. Here we test these predictions by computing different indices of within and between population diversity, mainly using a dataset obtained merging original and published data on the Hill Tribes. This larger Hill Tribes dataset allowed us to analyse a total of 10 populations. In fact, a preliminary analysis with all the 12 samples available (including both the samples we collected and those of Ref. [5]) indicated the absence of genetic differentiation between two White Karen and between two Lahu populations, which were therefore pooled bringing the total number of populations to 10. Here and in the following we will use the term *tribe *to refer to the 6 major ethnic groups (the Hill Tribes) and *populations *to indicate the 10 different samples.

#### Within-population genetic diversity in 6 populations (original data)

**Figure 1:**
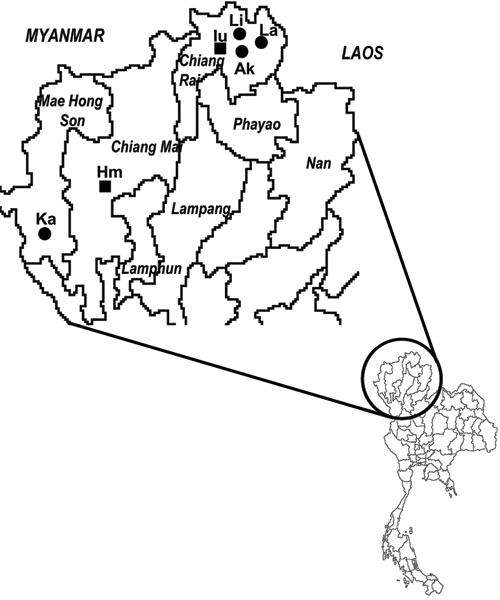
**Geographic location in Thailand of the samples typed in this study**. White Karen (Ka), Hmong (Hm), Iu-Mien (Iu), Lisu (Li), Lahu (La), Akha (Ak). Filled squares: Hmong-Mien speaking tribes; filled circles: Sino-Tibetan speaking tribes.

The analysis of 380 bp of the mtDNA control region in 137 individuals from the 6 populations typed in this study (Figure 1) identified 66 different haplotypes. Only 10 haplotypes were shared between two or more populations. Within population, diversity values are very high in all tribes, with the exception of the matrilocal White Karen tribe (Table 1). The Y-chromosome STRs were typed in a subset of 79 individuals, obtaining 64 different haplotypes. None of the Y-chromosome haplotypes was observed in more than one population. Comparably smaller values of diversity are observed in two Sino-Tibetan patrilocal tribes, Akha and Lisu (Table 1). Raw data are available from the authors on request.

**Table 1:** Social structure, linguistic family and genetic diversity in the Hill Tribes

Social structure	Population	Language family^a^	mtDNA (n)	k	h	π	Y-STRs (n)	k	h	π
			380 bp				15 loci			
Matrilocal	White Karen	ST	31	8	0.735	6.09	19	16	0.983	6.91
	Lahu	ST	25	20	0.980	7.73	15	15	1.000	9.25
										
Patrilocal	Akha	ST	26	16	0.920	6.34	14	7	0.868	4.40
	Lisu	ST	20	11	0.920	6.94	11	7	0.891	6.18
	Hmong	HM	14	9	0.934	7.42	7	7	1.000	6.52
	Iu-Mien	HM	21	15	0.938	7.38	13	12	0.987	8.70

			304 bp				6 loci			
Matrilocal	Red Karen^b^	ST	39	11	0.831	3.88	30	14	0.931	3.57
	White Karen^c^	ST	71	16	0.808	4.37	39	20	0.919	3.10
	Lahu^c^	ST	64	26	0.918	6.01	32	25	0.972	3.89
										
Patrilocal	Akha	ST	26	16	0.920	5.55	14	4	0.571	1.38
	Akha^b^	ST	91	24	0.932	5.12	21	5	0.352	0.55
	Lisu	ST	20	11	0.916	6.36	11	7	0.891	2.78
	Lisu CR^b^	ST	53	26	0.923	5.12	9	7	0.944	3.22
	Lisu MHS^b^	ST	42	19	0.955	5.73	22	9	0.883	3.19
	Hmong	HM	14	9	0.934	6.31	7	6	0.952	3.09
	Iu-Mien	HM	21	15	0.938	5.97	13	12	0.987	3.85

Samples description, number of individuals typed for mitochondrial and Y-chromosomal DNA, and standard indices of within-population genetic diversity. The first half of the table refers to the original data typed in this study. The second half of the table refers to the joint dataset (original + published data) used in most analyses.

k: number of haplotypes; h: gene or haplotype diversity; π: mean number of pairwise differences ^a^ST = Sino-Tibetan, HM Hmong-Mien; ^b ^data only from Oota et al. [5]; ^c ^data from Oota et al. [5] and present work.

#### Within-population genetic diversity in 10 populations (original + published data)

**Figure 2:**
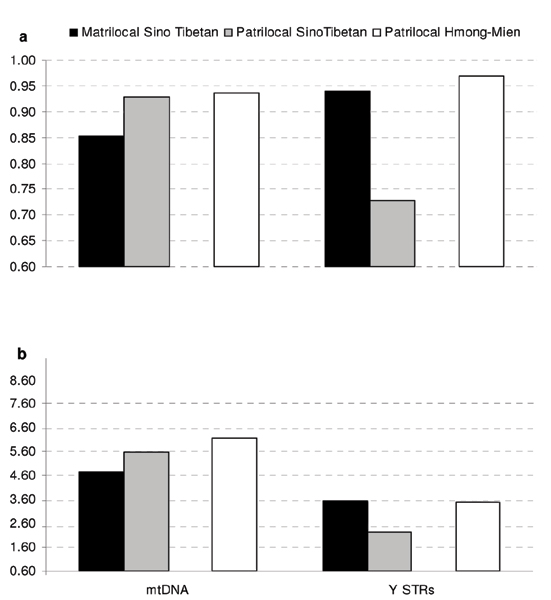
**Histograms of genetic diversity within the Hill Tribes**. Standard indices of genetic diversity within Hill Tribes populations. Average values of gene diversity (**a**) and average mean number of pairwise differences (**b**) for mtDNA and Y-chromosome STRs in samples grouped by social and linguistic criteria.

The results obtained with the larger dataset are now analysed in detail, with patrilocal tribes subdivided by linguistic family (Sino-Tibetan and Hmong-Mien; all matrilocal tribes belong to the Sino-Tibetan family and cannot be subdivided). Specific patterns are observed at different markers as predicted by the social structure, with an interesting additional feature not observed in earlier studies (Table 1 and Figure 2).

On the average, matrilocal populations are less variable than both Sino-Tibetan and Hmong-Mien patrilocal ones at mtDNA, regardless of the diversity index we consider. Within patrilocal tribes, Hmong-Mien are very similar, or slightly more variable (for the mean number of pairwise differences) than Sino-Tibetans. When male-transmitted Y STRs are analysed, a significantly higher genetic variation is observed, as expected, in matrilocal populations within the Sino-Tibetan family. Hmong-Mien patrilocal groups, however, show a genetic diversity very similar to the matrilocal samples. In other words, social and linguistic factors seem to interact since low variability at Y chromosomes is observed in patrilocal Sino-Tibetans but not in patrilocal Hmong-Mien tribes. The pattern of higher variability in the Hmong-Mien group appears therefore unrelated to the social structure: if patrilocal Hmong-Mien and matrilocal Sino-Tibetan populations were compared (for examples, being the patrilocal Sino-Tibetan samples unavailable), the relationship between social structure and genetic variation at different markers would not have emerged. We note that i) similar inferences are supported by the results obtained analysing only our original data, which includes fewer populations but more markers (previous section, see Table 1), and ii) either Student *t *or Mann-Whitney *U *tests (or both) provide statistical support for most of, but not all, the differences described above; the general trend is however clear, and since very few populations are included in each of the defined category, we expected that the typical statistical significance with P < 0.05 can not be always reached.

#### Between-population genetic diversity in 10 populations (original + published data)

The results obtained with the AMOVA analyses using different grouping schemes (numbered from 1 to 8) are reported in Table 2. In general, populations are statistically differentiated both at mtDNA and Y-chromosome markers in all the analyses, whereas differently defined groups of populations are never so. Population divergence ranges between 5% and 14% at mtDNA sequences, and between 5% and 36% at Y- chromosomes STRs, thus confirming that the tendency to migrate is higher in women than in men [1]. Each set of AMOVA analysis is considered in the following.

**Table 2:** Results obtained using 8 different AMOVA (Analysis of Molecular Variance) schemes

			mtDNA		Y STRs	
	
	n. of populations	n. of groups	Φst	Φct	Φst	Φct
1. Matrilocal tribes	3	1	0.14***	-	0.05***	-
2. Patrilocal tribes	7	1	0.06***	-	0.36***	-
3. Sino-Tibetan patrilocal tribes	5	1	0.06***	-	0.33***	-
4. Hmong-Mien patrilocal tribes	2	1	0.05*	-	0.13*	-
5. Patrilocal tribes, Sino-Tibetan vs. Hmong-Mien	7	2	0.06***	0.0	0.34***	-0.05
6. All tribes, Sino-Tibetan vs. Hmong-Mien	10	2	0.09***	0.0	0.17***	-0.01
7. Sino-Tibetan tribes, matrilocal vs. patrilocal	8	2	0.09***	-0.01	0.18***	-0.03
8. All tribes, matrilocal vs. patrilocal	10	2	0.09***	0.0	0.17***	-0.04

The results obtained with AMOVA assuming different groups of populations, or different hierarchical levels, are provided as *Φ*_*st *_(index of divergence between populations) and *Φ*_*ct *_(index of divergence between groups) statistics. ***p < 0.001; *p < 0.05.

##### AMOVA schemes 1–2

mtDNA genetic distances among matrilocal tribes are higher than those obtained among patrilocal tribes (14% and 6% respectively). The opposite patterns, with even more extreme differences, are found in the analysis of Y-chromosomes: patrilocal populations are highly differentiated among them (*Φ*_*st *_= 36%), whereas matrilocal ones are rather homogeneous (*Φ*_*st *_= 5%). Again, these figures support the view that postmarital residence habits produce patterns of sex-specific gene flow.

##### AMOVA schemes 3–4

When the analysis was performed separately for the different linguistic families in the patrilocal tribes (no such subdivision is possible in matrilocal tribes), we found, especially at the Y-chromosome markers, that Sino-Tibetan tribes are more differentiated among them than Hmong-Mien. Even if the geographic distance between Hmong and Iu-Mien (the two Hmong-Mien speaking tribes in our dataset) is much higher than the average distance between the 5 Sino-Tibetan patrilocal tribes (2 Akha and 3 Lisu), they clearly appear genetically more homogeneous.

##### AMOVA schemes 5–6

When the two groups of Sino-Tibetan and Hmong-Mien speaking population are compared, either including all or only the patrilocal tribes, the AMOVA index *Φ*_*ct *_is never significantly different from 0, regardless of the marker considered. In other words, tribes are genetically differentiated (*Φ*_*st *_is significant) but the average level of divergence is not increased when tribes speaking languages of different families are compared.

##### AMOVA schemes 7–8

Finally, we compared the two groups of populations defined on the basis of their social structure, i.e. matrilocal versus patrilocal tribes. As for the comparison between linguistic groups (schemes 5–6), the AMOVA results seem to suggest that the level of divergence between tribes is not enhanced by differences in social habits (i.e., *Φ*_*ct *_is not statistically different from 0). The average divergence between a patrilocal and a matrilocal tribe is therefore similar to the average divergence between pairs of populations within the two groups, the latter being actually a mean of the very different divergence values observed among patrilocal and matrilocal tribes (see the AMOVA schemes 1 and 2).

### Estimates of migration rates

Gene flow rates estimated in different comparisons are reported in Table 3. Although the errors of the likelihood estimates between specific pairs of populations are quite variable, as commonly observed in single locus analyses [12,13], several trends clearly emerge from our analyses based on averages rates or pooled samples.

**Table 3:** Migration rates estimated by MIGRATE

	mtDNA	Y-Chrom
1. Between two populations belonging to the same Sino-Tibetan matrilocal tribe	2.4	4.1
2. Between two populations belonging to different Sino-Tibetan matrilocal tribes	6.1	4.2
Average	4.25 (0.98)	4.05 (0.45)

3. Between two populations belonging to the same Sino-Tibetan patrilocal tribe	19.0	2.2
4. Between two populations belonging to different Sino-Tibetan patrilocal tribes	8.2	0.7
5. Between two populations belonging to different Hmong-Mien patrilocal tribes	14.2	2.4
Average	13.80 (1.52)	1.76 (0.23)

Estimates of the rate of gene flow (*θ *= *N*_*e*_*m*,) obtained in different population comparisons. Comparisons 1–2 refer to matrilocal tribes, from the same (1: White Karen vs. Red Karen) or different (2: pooled Karen vs. Lahu) ethnic groups. Comparisons 3–5 refer to patrilocal tribes, from the same (3: average between Akha vs. Akha and Lisu vs. Lisu) or different (4: pooled Akha vs. pooled Lisu) ethnic groups within the Sino-Tibetan linguistic family, and from different ethic groups within the Hmong-Mien linguistic family (5: Hmong vs. Iu Mien). Standard errors based on different runs and different pairwise estimates within the same group of comparisons are reported in parentheses.

i) On the average, about 2 men and 14 women are exchanged in patrilocal tribes every generation, respectively, and about 4 men and 4 women do the same in matrilocal tribes every generation. Therefore, whereas male and female migration rates appear similar in matrilocal tribes, fewer males, but much more females, immigrate in patrilocal compared to matrilocal tribes.

This result is consistent with our analyses on the genetic variation within populations (see above) and with previous studies based on different methods and partially different data [5,6]: maternal and paternal gene flow rates follow an opposite pattern in patrilocal and matrilocal tribes, and migration control is in tighter in patrilocal than in matrilocal groups. It is reasonable to conclude that restriction to female migration in matrilocal communities results in similar dispersion rates for males and females. Conversely, in patrilocal communities the generally lower tendency of males to migrate would be further enhanced by strict social rules. Our point estimates suggest higher migration rates for both men and women than previously estimated [6], especially in patrilocal groups (between 2 and 4 times higher). Confidence intervals across studies, however, overlap.

ii) A reasonable expectation about gene flow patterns is that different Hill Tribes (belonging to the same linguistic family) exchange fewer individuals than different populations within a tribe. We observe this reduction only in patrilocal (analysis 3 vs. analysis 4) but not in matrilocal (analysis 1 vs. analysis 2) groups. Again, we believe that this result should be interpreted as evidence for the tighter migration control in patrilocal than in matrilocal societies.

iii) When the comparison between different linguistic families is possible, i.e. being equal all the other factors (analysis 4 vs. analysis 5 in Table 3), Hmong-Mien speaking tribes seem to show a higher tendency to migrate. However, when we consider separately the migration rates between all pairs of patrilocal populations (results not reported) this conclusion should be re-evaluated. In fact, Iu-Mien, a Hmong-Mien tribe, shows high and similar female migration rates both with Hmong-Mien and with Sino-Tibetan populations, whereas Akha (a Sino-Tibetan tribe) shows low and similar male migration rates both with Sino-Tibetan and Hmong-Mien populations. In other words, the observed differences between different linguistic groups seem more related to single tribe effects than to a language – related component.

### The genetic affiliation of the Hill Tribes

Here we tested with two large mtDNA and Y-chromosome databases the possible genetic affinities of the Hill Tribes, and thus their possible origin, using the Bayesian clustering method implemented in BAPS.

#### mtDNA dataset (99 populations)

The global mtDNA dataset we assembled includes 99 populations and 3644 individuals (see Additional File 1). 1386 different haplotypes, 930 of them found only in a single individual, were identified. The most likely partition inferred by BAPS supports the presence of 68 groups, 51 including only one population. This clustering solution was not informative to identify the most likely Hill Tribes genetic neighbours. Therefore, as in several other studies of clustering [14,15], we run the analysis with increasing number of clusters, starting from the smallest value, K = 2, up to K = 5. A maximum of K = 5 was chosen because for K > 5 almost only single-population groups were additionally identified.

In general, the BAPS partitions significantly reflect the linguistic affiliation of the populations (tested by partial correlation Mantel tests as described in the Methods, p < 0.001 for K = 2 to 5). On the contrary, geographically close populations are included by BAPS in the same cluster more frequently than expected by chance only for larger K values (p > 0.05 for K = 2 or 3; p < 0.001 for K = 4 or 5). Significant correlations coefficients range between 0.15 and 0.25.

**Figure 3:**
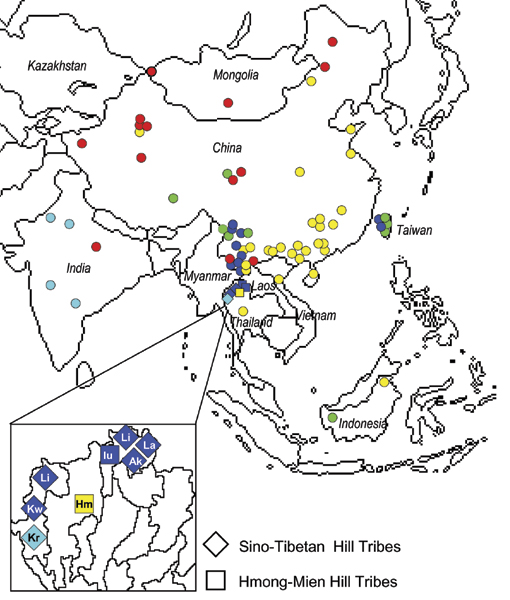
**Results provided by the BAPS analysis: mtDNA**. Five clusters of populations are indicated with different colours.

Analysing more in detail the partition for K = 5 (Figure 3), we can identify i) a *Northern Altaic *clade (red circles) in Northern China and Mongolia, which includes also three Southern China samples (Yunnan) and one sample form India; ii) an *Indian Indo-European *clade (in light blue), which includes all but one Indian samples, and the Red Karen Hill Tribe; iii) a *Central-Southern Sino-Tibetan *clade (blu circles) which includes Yunnan populations, most Hill Tribes, and three Taiwanese populations; iv) an *Eastern *clade (yellow circles) which includes all Hmong-Mien speaking populations (with the only exception represented by the Iu-Mien Hill Tribe), and several Sino-Tibetan and Tai-Kadai populations; v) a more heterogeneous clade (green circles) which includes Central and South-Eastern populations speaking either Sino-Tibetan or Austronesian languages.

Most of the Hill Tribes in Thailand seem to preserve a maternal genetic legacy with their likely geographic origin.

Lahu, Lisu and Akha are Sino-Tibetan populations of Lolo extraction, and some historical record suggest that they established in Yunnan in ancient times (starting 2000 years ago) from the Tibet region, and then migrated trough Myanmar in Northern Thailand about 100–200 years ago [16]. Consistently with this hypothesis, all of them are attributed to the *Central Southern Sino-Tibetan *genetic clade in the BAPS analysis, mainly located in the Yunnan region.

The Karen people belong also to the Sino-Tibetan language family, but to a different linguistic branch, the Karenic. Their ethnic origin is largely unknown, since Karen usually avoid contacts with other groups, and leave therefore no traces in the regions they pass through during the migrations. The characteristics of the Karen suggest China, near Tibet, as a possible origin, but none of them live there today [16]. From there, they entered Myanmar around the sixth-seventh century AD, where they still live in large numbers (> 2 millions). Some groups then migrated to Thailand more recently. Unfortunately we do not have genetic data for the Myanmar groups, but, at least for the White Karen, their genetic affiliation with the *Central Southern Sino-Tibetan *clade in the BAPS analysis is consistent with their hypothetical place of origin in Tibet/China regions. On the other hand, the Red Karen in our dataset cluster within the *Indian Indo-European *genetic clade. This result is difficult to interpret, and may be simply due to the limited sample sizes or to the lack of significant reference samples. We note however that the ethnic origin of Red Karen is even more debated, with some authors suggesting that they are Mon-Khmer people (which belong to a non Sino-Tibetan linguistic family, the Austro-Asiatic) who adopted a Karenic language [16].

Finally, the two Hmong-Mien speaking Hill Tribes in our data, the Hmong and the Iu-Mien, are classified in the *Eastern *and the *Central Southern Sino-Tibetan *genetic clades, respectively. The heartland of the Hmong people is considered Kweichow, a Chinese province Eastern of Yunnan, where they established at least 2000 years ago probably arriving from more Eastern areas [16]. Migrations into Thailand through Laos are documented since the second half of the nineteenth century. The BAPS maternal affiliation of Hmong Hill Tribes in Thailand confirms their origin and genetic legacy with South-Eastern China. On the other hand, Iu Mien, whose origin is located as for Hmong people in South Eastern China from which they started to migrate southwards to Vietnam in the thirteenth century entering Thailand about 200 years ago, are genetically affiliated with the *Central Southern Sino-Tibetan *group. This contrasting, but interesting result, may have an explanation in the social habits of the Iu-Mien, who adopt children from neighbour communities to enlarge their work force [16,17]. According to the literature, the percentage of adopted individuals under the age of 20 corresponds to about 20% of the population. The genetic composition of the Iu-Mien people in our dataset may be therefore mixed, resulting in their genetic affiliation with the surrounding Hill Tribes belonging to the *Central Southern Sino-Tibetan *clade.

#### Y-chromosome dataset (53 populations)

**Figure 4:**
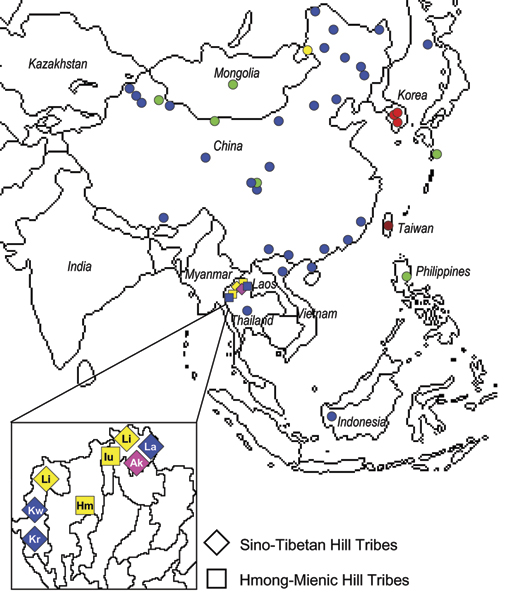
**Results provided by the BAPS analysis: Y chromosome**. Six clusters of populations (the most likely partition identified by BAPS) are indicated with different colours.

A dataset of 53 populations and 3044 individuals was assembled (see Additional File 1). 1204 different haplotypes, 723 of them found only in a single individual, were identified for the 6 loci overlapped across studies. The clustering method we applied inferred a partition with 6 groups as the most likely (see Figure 4). Unfortunately, genetic data are missing from several geographic regions important for our purposes, and the vast majority of the samples are included in a single, geographically widespread, BAPS-defined clade (in blue in Figure 4). This dataset is therefore non-informative to identify the genetic legacy and origin of the Hill Tribes, but the results we obtained suggest that: i) China appears less structured at the Y-chromosome markers compared to the mtDNA, but this may be the simple consequence of the small number, possibly homoplasic, microsatellite loci available for the Y-chromosome analyses; all matrilocal Hill Tribes are assigned to this group, possibly as a consequence of their higher male permeability; ii) one private and one almost private (with the odd exception of a Mongolian Han sample from Northern China) clades are identified for the patrilocal Hill Tribes. The presence of two independent Y-chromosome clades in the Hill Tribes can be attributed to the lack of reference populations in the database. An alternative explanation might be a specific drift effect at Y-chromosomes in the patrilocal tribes, where male exchanges are restricted.

## Conclusion

The Northern Thailand Hill Tribes show a high level of population structuring. In a restricted geographic area, the levels of divergence can reach the values observed in world wide analyses [18]. Most of tribes, with an exception related to a specific habit of children adoptions, preserve traces of genetic similarity with the populations living in the areas (in different countries) where the geographic origin of these groups has been suggested.

The major factor explaining the differences at mtDNA and Y-chromosome markers between tribes is confirmed to be the difference between postmarital habits of residence, with social rules more strictly enforced in patrilocal than in matrilocal tribes. Overall, the migration rates we found for the Hill Tribes are higher than previously estimated with a different method on a smaller dataset.

Linguistic differences, which clearly play a role when a larger Asian region in considered, have limited effects at this microgeographic scale. However, specific situations that may disrupt the otherwise robust relationship between social structure and sex specific genetic diversity, related to linguistic or population-specific traits, are identified and should be considered in future studies.

## Methods

### Populations, samples, and markers

At least 10 Hill Tribes can be identified in Northern Thailand, but 6 of them are regarded also by the Thai Government as the main groups: Karen, Hmong, Iu-Mien (Yao), Lisu, Lahu, and Akha. Blood samples were collected with informed consent from all these six groups. In particular, the geographic locations of the samples (see Figure 1) are as follow: Mae Swan Noi village, Mae Hao sub-district, Mae Sarieng district, Mae Hong Son province (White Karen); Mae Khi village, Pong Yang sub-district, Mae Rim district, Chiang Mai province (Hmong); Huai Mae Sai village, Mae Yao sub-district, Muang district, Chiang Rai province (Iu-Mien); Acha village, Mae Yao sub-district, Muang district, Chiang Rai Province (Akha); Tha Kao So village, Mae Khao Thom sub-district, Muang district, Chiang Rai province (Lahu); Wiang Klang village, Mae Khao Thom sub-district, Muang district, Chiang Rai province (Lisu).

The social structure (matrilocality or patrilocality) and the language family (Sino-Tibetan or Hmong-Mien) of each tribe are reported in Table 1.

DNA was extracted following standard procedure. Each sample was typed for maternally and paternally inherited markers. We sequenced 380 base pairs of the first hypervariable region of mtDNA from position 16037 to 16416 following the standard method described by Schurr et al. [19]. 15 Y-chromosome STRs (DYS19, DYS388, DYS389i, DYS389ii, DYS390, DYS391, DYS392, DYS393, DYS426, DYS436, DYS437, DYS439, Y-gata-A7.1, Y-gata-A7.2, Y-gata-A10) were typed as previously described [20-22].

A joint dataset for the Hill Tribes was assembled integrating our data with the data from Oota et al. [5]. Similarly, a reference Asian database was assembled for both the mtDNA sequences (99 populations) and the Y-chromosome microsatellites (53 populations). All the populations and the relative bibliographic references used for this large database are reported in the Additional File 1. The analyses on the joint datasets required a reduction of the markers to the available overlapping regions or loci: mtDNA sequence stretch was reduced from 380 bp to 304, and six Y-chromosome microsatellites were considered.

### Statistical analyses

Genetic variation within groups was estimated using gene or haplotype diversity [23], and the mean number of pairwise differences [24]. Genetic distances between populations were estimated using *Φ *statistics, analogues of Wright's F statistics that additionally take into account the evolutionary distance between individual haplotypes [25]. Pairwise differences between haplotypes and *R*_*st*_, a molecular version of *F*_*st *_under the stepwise-mutation model [26] were used for mtDNA and Y-STRs, respectively. The genetic structure was estimated assuming different schemes in the analysis of molecular variance (AMOVA, [25]), where genetic diversity is partitioned in up to three levels: within population, among populations within groups, and among groups. Groups were defined on the basis of the linguistic or social structure affiliation. The software ARLEQUIN 3.1 [27] was used to compute genetic diversity indices, genetic distances, and AMOVA.

Male and female gene flow rates between populations were estimated as the number of migrant per generation, *N*_*e*_*m*, using the maximum likelihood method implemented in the software MIGRATE [28]. *N*_*e *_is the effective population size, *m *is the migration rate. The Brownian motion approximation of the stepwise mutation model was applied to Y-chromosome microsatellites. Migration rates between pairs of populations were assumed symmetrical, and were estimated as averages across three independent runs. Each runs included 10 short chain (10000 or 100000 genealogies per chain for the Y-chromosome microsatellites and the mtDNA sequences, respectively) and 3 long chains (100000 or 1000000 genealogies per chain depending on the marker), all with increments of 20 and 200 steps. The final estimates of *N*_*e*_*m *were obtained as averages across runs.

In the analysis of the assembled Asian database, the Bayesian approach implemented in BAPS [29,30] was used to infer homogenous groups of populations. The stability and convergence of the analysis were ensured by considering five replicates of the simulation runs. The attribution of each Hill Tribes population to a specific group inferred by BAPS was taken as evidence for their genetic similarity, and possibly common origin, with these groups.

Using the results produced by BAPS, we additionally tested in the large databases (by means of Mantel tests [31] using the software PASSAGE [32]) the correlation between genetic, linguistic and geographic distances. The matrix of genetic distances was simply defined with 0/1 values, assuming a distance of 0 between samples assigned by BAPS to the same cluster and 1 between samples from different clusters. In the matrix of language distances, four different values were assumed, from 3 (in the comparison between groups speaking languages of different families) to 0 (in the comparison between localities where the same language was spoken), with intermediate values assigned on the basis of the hierarchical classification of languages reported in Ethnologue [33]. Spherical distances were used for the geographical matrix.

## Competing interests

The authors declare that they have no competing interests.

## Authors' contributions

DB performed the statistical analyses, assembled the databases and helped to draft the manuscript. SF participated in statistical analyses and in writing the manuscript. MS and JK performed the molecular typing. LC contributed to assembling the mitochondrial DNA database. CTS gave a substantial contribution to the Y-chromosome database collection before its publication. MS was responsible for the Y-chromosome laboratory analysis. DK was responsible for sample collection and participated in study design and coordination. GB designed and coordinated the study and wrote the manuscript. All authors read and approved the final manuscript.

## Supplementary Material

Additional file 1 **Summary of the data used in the Asian databases**. The Excel file *AdditionalFile_Besaggio_et_al.xls *includes name, geographic location, sample size, language and bibliographic references for each population used in the BAPS analyses. Click here for file
